# Generation of OAM beams with quasi omnidirectional pattern using simple slotted waveguide array

**DOI:** 10.1038/s41598-024-57039-5

**Published:** 2024-03-18

**Authors:** Yuvraj B. Dhanade, Amalendu Patnaik

**Affiliations:** Department of Electronics and Communication Engineering, Indian Institute of Technology Roorkee, Roorkee, 247667 India

**Keywords:** Electrical and electronic engineering, Electronic and spintronic devices

## Abstract

Theory and design aspects of the Slotted Waveguide Antenna (SWA) array for the simultaneous generation of Orbital Angular Momentum (OAM) modes are proposed in this paper. The proposed method is a novel approach to OAM generation that uses a simple feeding scheme and has better performance, especially in terms of mode purity. Due to its all-metallic structure, the high-power OAM beams with first-order modes ($+1 \,  \&  \,-1$) can be generated simultaneously along the propagation axis but in opposite directions, which improves the coverage and makes the proposed SWA be used in applications like radar target detection by mounting it on a mechanically rotating platform. The proposed theory is verified by the electromagnetic (EM) simulations, followed by the experimental verification done on the laboratory prototype. The near-field characteristics of the proposed antenna confirm the generation of first-order OAM beams with high mode purity. The far-field characteristics show the high gain and quasi-omnidirectional radiation pattern. Transmission of high-power OAM beams with good far-field characteristics makes the proposed antenna mitigate the issues of long-range OAM communication to a certain extent. However, the proposed antenna can be served well in strategic applications where the mostly high-power microwave is involved.

## Introduction

The theory of electrodynamics proves that electromagnetic (EM) waves carry not only energy but also momentum: linear and angular when traveling in a far zone^1^. The angular momentum can be further classified into Spin Angular Momentum (SAM) and Orbital Angular Momentum (OAM) related to the polarization and spatial phase distribution of the EM waves, respectively. In contrast to SAM, which has only two operating modes, OAM can theoretically have infinite modes of operation. Because of the vorticity in the OAM beams, all the operating modes are orthogonal to each other and can be multiplexed together on the same frequency with less crosstalk in between^2^. This can provide a new degree of freedom in wireless communication and can substantially alleviate the scarcity in the radio spectrum, thus improving the spectrum efficiency. Data capacity improvement in wireless communication by multiplexing different OAM modes has already been proven^3^. Because of its property called “ phase singularity”, the OAM beams have excellent advantages in detection and imaging, especially for rotating targets^4,5^. It has been proved that the radar target detection/imaging can be performed more efficiently using OAM beams than plane waves^6^. Also, the outdoor experimental study proves that the single mode OAM beam can be used to reconstruct the target with greater resolution^7^. Moreover, to reduce the system’s complexity in the case of target imaging, various algorithms are being developed to perform the imaging with one or two OAM modes^8,9^. In addition, OAM beams can also be used for highly secured wireless communication systems for strategic applications^10^.

OAM beams in the radio domain can be generated using phase modulation method and/or special antenna radiation method. In the phase modulation method, the mask of precise dimensions is placed in front of the antenna transmitting plane wave to generate the desired OAM mode. In this case, Spiral Phase Plate (SPP) is mostly used as a mask to generate the OAM beam^11^. However, Holographic Plates (HP)^12^, reflectarray^13^, and metasurfaces^14^ are also being used as a mask for the OAM generation. But once the antenna profile is fixed with these masks, it can generate only a single mode. However, the mode purity of the generated OAM beams is poor. Under the special antenna radiation method, the antenna is designed to generate OAM beams directly. In this category, Uniform Circular Antenna Array (UCA) is the most widely used method to generate multiple OAM modes by providing equal amplitude and corresponding phase shifts to the antennas^15–20^. But the requirement of a complex feeding network consisting of power dividers, phase shifters, switches, etc., makes the overall structure complex and difficult to design. Characteristic Mode Theory (CMT) has also been adopted for the OAM generation using single antenna, but the generated OAM beams have less purity^21,22^. Besides these planar antennas, simple three-dimensional antenna structures are also used for high-power OAM generation^2,23^. But these antennas could generate only a single mode with their structure with less mode purity. A cassegrain reflector antenna for the simultaneous generation of ($0, +1, -1$) OAM modes is proposed^24^, but the generated OAM beams seems to have less mode purity. However, the structure is very bulky and complex to design. Recently, a study on corrugated cylindrical waveguides has been conducted for millimeter waves, showing that the hybrid mode in the corrugated waveguide carries well-defined OAM characteristics^25^.

In this paper, a novel slotted waveguide antenna array has been designed to simultaneously generate first-order OAM beams. Most of the OAM antennas available in the literature are dielectric-based planar antennas which are limited to be utilized in high-power microwave applications. However, there are many applications where all-metallic antennas that can transmit high-power microwave signals are needed. One such application of the OAM beams is radar target detection/imaging where such antennas, e.g. proposed SWA can be utilized. The proposed SWA has a simple structure operating at 10 GHz for X-band applications. It is made by cutting conventional simple rectangular slots in the broad walls of a WR-90 rectangular waveguide. This paper provides a new approach to OAM generation that uses a simple feeding scheme bearing no phase shifter in contrast to UCAs requiring complex feeding networks. The theory of first-order OAM generation using the proposed structure is deduced and verified experimentally by performing near-field and far-field measurements. Moreover, because of its simple feeding scheme with no phase shifters the purity of the generated OAM beams is relatively high as compared to the other related prior art.

**Figure 1:**
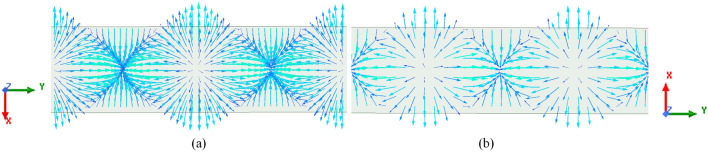
Simulated surface current distribution for, (**a**) top broad wall and, (**b**) bottom broad wall of a rectangular waveguide for $TE_{10}$ mode.

## Results

### Theory of first-order OAM beam generation using SWA.

Generating OAM beams with particular modes is a phase-dependent phenomenon, and in the radio domain, it is mainly achieved using UCA. In general, the EM beam carrying OAM can be mathematically represented in a cylindrical coordinate system as,

1 $$\begin{aligned} E_{OAM}(\rho , \phi , z) = E_{l}(\rho , z)e^{jl\phi } \end{aligned}$$

where, $E_{l}(\rho , z)$ is the amplitude and $e^{jl\phi }$ is the helical phase distribution of the OAM beam for a particular mode, *l*. In Eq. (1), the transverse azimuth angle $\phi$ is a periodic function mainly responsible for the OAM generation. In UCAs, this periodicity can be achieved by placing the antenna elements circularly and the corresponding mode can be generated by incorporating the appropriate phases. In order to generate the OAM beams with an N-element UCA the progressive phase shift, $\Delta \phi =\frac{2\pi l}{N}$ should be introduced between the adjacent elements with a maximum tolerable phase shift error of $22.5^{\circ }$^26^. So to generate first-order ($l=\pm 1$) OAM beams with four antenna elements, all the elements has to be fed such that there should be the progressive phase shift of $\Delta \phi =\mp 90^{\circ }$ between the adjacent elements. This analogy can be used in the slotted waveguides in order to generate OAM beams. For this purpose, the surface current distribution on the broad wall should be studied as the phase shift requirement between the adjacent radiators (i.e., slots) can be achieved by precisely locating them concerning the surface current distribution.

**Figure 2:**
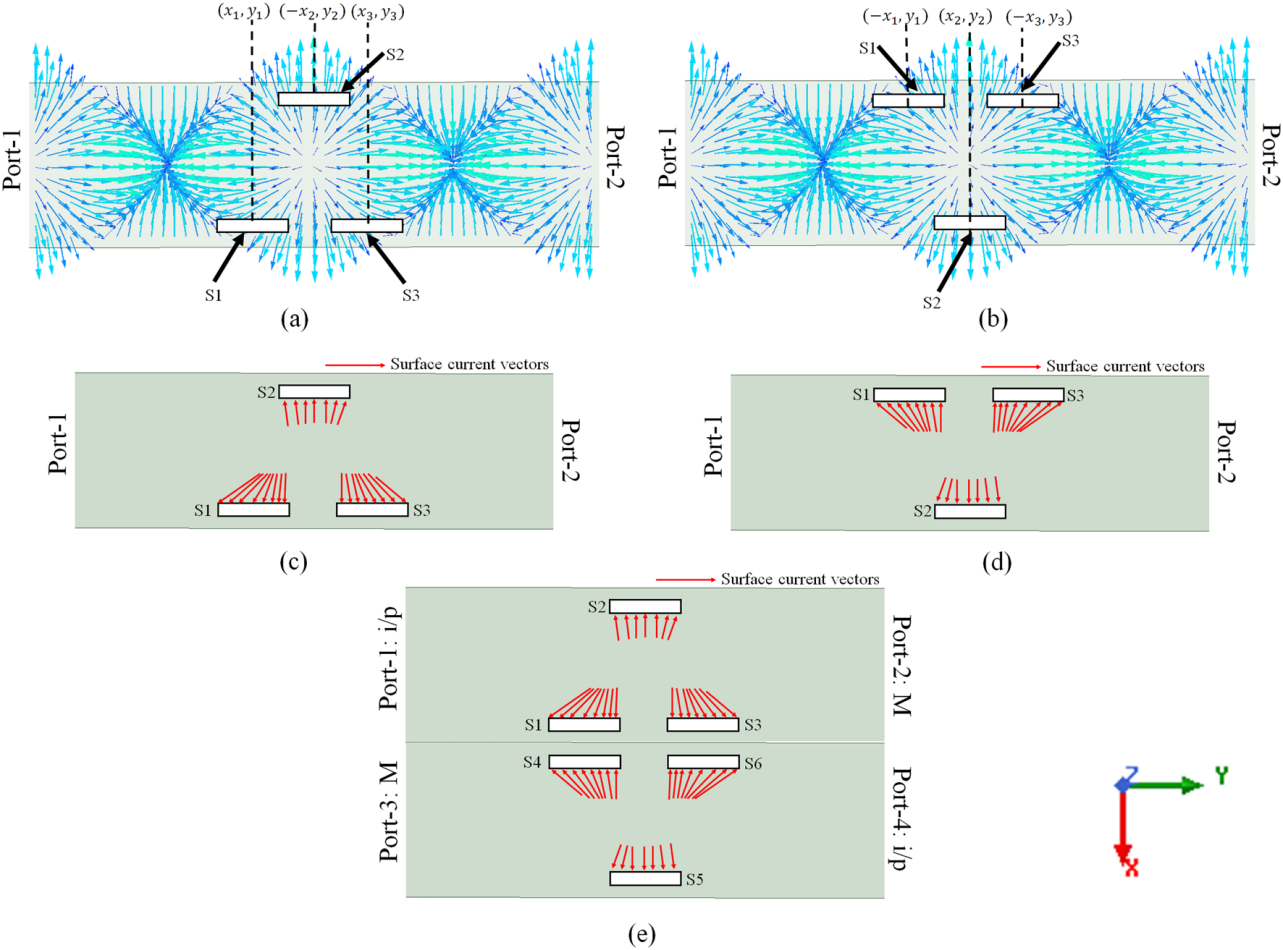
Mechanism of first-order OAM beam generation using SWA, (**a**) three rectangular slots placed $\lambda _{g}/4$ apart in the top broad wall, (**b**) swapping of slots’ positions, i.e., slots in the upper-half are shifted to lower-half of a broad wall and vice versa, (**c**) representation of surface current vectors exciting slots in (figure **a**), (**d**) representation of surface current vectors exciting slots in (figure **b**) and, (**e**) SWA array for the first-order OAM generation.

We use the concept of SWA to generate OAM beams by correctly optimizing the slots’ positions in the broad wall to provide the appropriate phase shifts between them. The traditional rectangular slots are used for this study. When the standard rectangular waveguide is excited by fundamental $TE_{10}$ mode, then the surface current ($\mathbf {J_{s}}$) flowing on the broad wall is proportional to^27^,

2 $$\begin{aligned} {({\textbf{J}}_{{\textbf{s}}})_{U}} \propto \cos \bigg(\frac{\pi x}{a}\bigg) {{\hat{\textbf{a}}}_{x}} - j\frac{k a}{\pi }\sin \bigg(\frac{\pi x}{a} \bigg) {{\hat{\textbf{a}}}_{y}} \end{aligned}$$

3 $$\begin{aligned} {({\textbf{J}}_{{\textbf{s}}})_{L}} \propto -\cos \bigg(\frac{\pi x}{a} \bigg) {{\hat{\textbf{a}}}_{x}} + j\frac{k a}{\pi }\sin \bigg(\frac{\pi x}{a} \bigg) {{\hat{\textbf{a}}}_{y}} \end{aligned}$$

where, Eqs. (2) and (3) correspond to the surface current on the top and bottom broad wall, respectively. Here, ${{\hat{\textbf{a}}}_{x}}$ and ${{\hat{\textbf{a}}}_{y}}$ are unit vectors in $x-$ and $y-$directions, respectively, *a* is the broad wall length and, $k=2\pi /\lambda _{g}$ is the wavenumber in the waveguide, where, $\lambda _{g}$ is the guided wavelength. So according to Eqs. (2) and (3), the current distribution on the surface of the broad wall of a rectangular waveguide for $TE_{10}$ mode is as shown in Fig. 1. Usually, the rectangular slots are placed in the broad wall to disrupt this current distribution in order to have radiation in the desired direction. Similarly, the EM beam bearing first-order OAM modes can be generated from these slots by placing them judiciously in the broad wall to fulfill the phase requirements in the case of OAM. This scenario is depicted in Fig. 2, which shows the step-by-step procedure of OAM generation using SWA.

Consider the top broad wall of a rectangular waveguide with its surface current distribution, as shown in Fig. 1a. The three rectangular slots say S1, S2, and S3 are placed in this broad wall at particular positions concerning the surface currents, the scenario is depicted in Fig. 2a. Here, (*x*, *y*) represents the positions of the center of the slots on the top broad wall. These slots have to be placed such that the horizontal distance between the adjacent slots is approximately equal to the quarter-guided wavelength or multiple of the quarter-guided wavelength, i.e., $y_{2}-y_{1}\approx m\lambda _{g}/4$, where, $m = 1, 2, 3, \ldots$. In this case, the $m=1$. The surface current vectors exciting these slots can be seen in Fig. 2c. As shown in the figure, the surface current vectors exciting S1 and S2 are almost $180^{\circ }$ out of phase. Because of this, the fields radiated by these two slots will be $180^{\circ }$ out of phase. But these slots i.e., S1 and S2 are at a distance of $\frac{\lambda _{g}}{4}$ introducing an additional phase shift of $-90^{\circ }$. Therefore, the total phase shift between the fields radiated by S1 and S2 is $90^{\circ }$. Similarly, with this analogy, the fields radiated by S2$\,  \&  \,$S3 will have $90^{\circ }$ phase shift, and S1$\,  \&  \,$S3 will have $180^{\circ }$ phase shift in between.

Now, if we swap the positions of the slots vertically, i.e., the slots in the upper half of a broad wall will be placed in the lower half and vice-versa, as shown in Fig. 2b. The corresponding surface current vectors exciting these slots are shown in Fig. 2d. In this case, the phase shifts between the adjacent and alternate slots are the same as in the case of Fig. 2a. Now take these SWAs in Fig. 2c and d to make the array, as shown in Fig. 2e. If Port-1 and Port-4 are excited, whereas Port-2 and Port-3 are matched terminated, the surface current vectors exciting the slots (S1 to S6) are shown in the figure. Now let’s analyze the possible phase distribution among all these slots for the scenario shown in Fig. 2e.

Let us take the slot S1 as reference and consider the phase of the field radiated by S1 to be $0^{\circ }$. Now as per the earlier discussion, if S1 is radiated with $0^{\circ }$ phase then S2 and S3 will be radiating with $-90^{\circ }$ and $180^{\circ }$ phases, respectively. Consider slots S3 and S6. It can be observed in Fig. 2e that the surface current vectors exciting these slots are out of phase introducing $180^{\circ }$ phase shift between them. But here, Port-1 and Port-4 are excited, so the path difference between S3 and S6 concerning their respective input ports is $\lambda _{g}/2$, which introduces the additional phase shift of $180^{\circ }$ between them. Therefore S3 and S6 radiate with the same phase. So the phase value of S6 can be considered as $180^{\circ }$. Similarly, slots S1 and S4 are in the same phase, so the phase value of S4 can be considered as $0^{\circ }$. Consider slot S5. The surface current vectors exciting the S5 are out of phase with S2, as shown in the figure. However, the path difference between S2 and S5 are the same concerning their input ports. So the total phase shift between S2 and S5 is $180^{\circ }$, hence the phase value of S5 can be considered as $90^{\circ }$. The phase of the fields radiated by all the slots are mentioned in Table 1.

**Table 1:** Theoretical phase values of E-field radiated by the slots.

Slot	S1	S2	S3	S4	S5	S6
${\text {Phase (}}{} \textit{l}={\text {-1)}}$	$0^{\circ }$	$-90^{\circ }$	$180^{\circ }$	$0^{\circ }$	$90^{\circ }$	$180^{\circ }$
${\text {Phase (}}{} \textit{l}={\text {+1)}}$	$0^{\circ }$	$90^{\circ }$	$180^{\circ }$	$0^{\circ }$	$-90^{\circ }$	$180^{\circ }$

All the slots shown in Fig. 2e seem to be placed in a quasi-circular orbit required to achieve periodicity in OAM and are radiating the fields with the corresponding phase values as shown in Table 1. The slots radiating with same phase i.e., S3 and S6 and S1 and S4, can be considered single element in pair. So this particular arrangement of the slots in the broad wall can be treated as four elements radiator. Moreover, there exist a phase shift of $90^{\circ }$ and $180^{\circ }$ between adjacent and alternate radiators, respectively, as required for the first-order OAM generation. Therefore an OAM beam with $l=-1$ mode can be generated by arranging the slots as shown in Fig. 2e. Similarly, if these slots are placed at same positions in the bottom broad wall of a rectangular waveguide, the polarity of the surface current vectors exciting these slots will be opposite, as shown in Fig. 1b. So, in this case, there will be a phase difference of $-90^{\circ }$ between the adjacent slots as shown in Table 1. Therefore, the OAM beam with $l=+1$ mode can be generated. To verify this theory, the EM simulations followed by the experimental measurements are accomplished which are discussed in the following sections.

**Figure 3:**
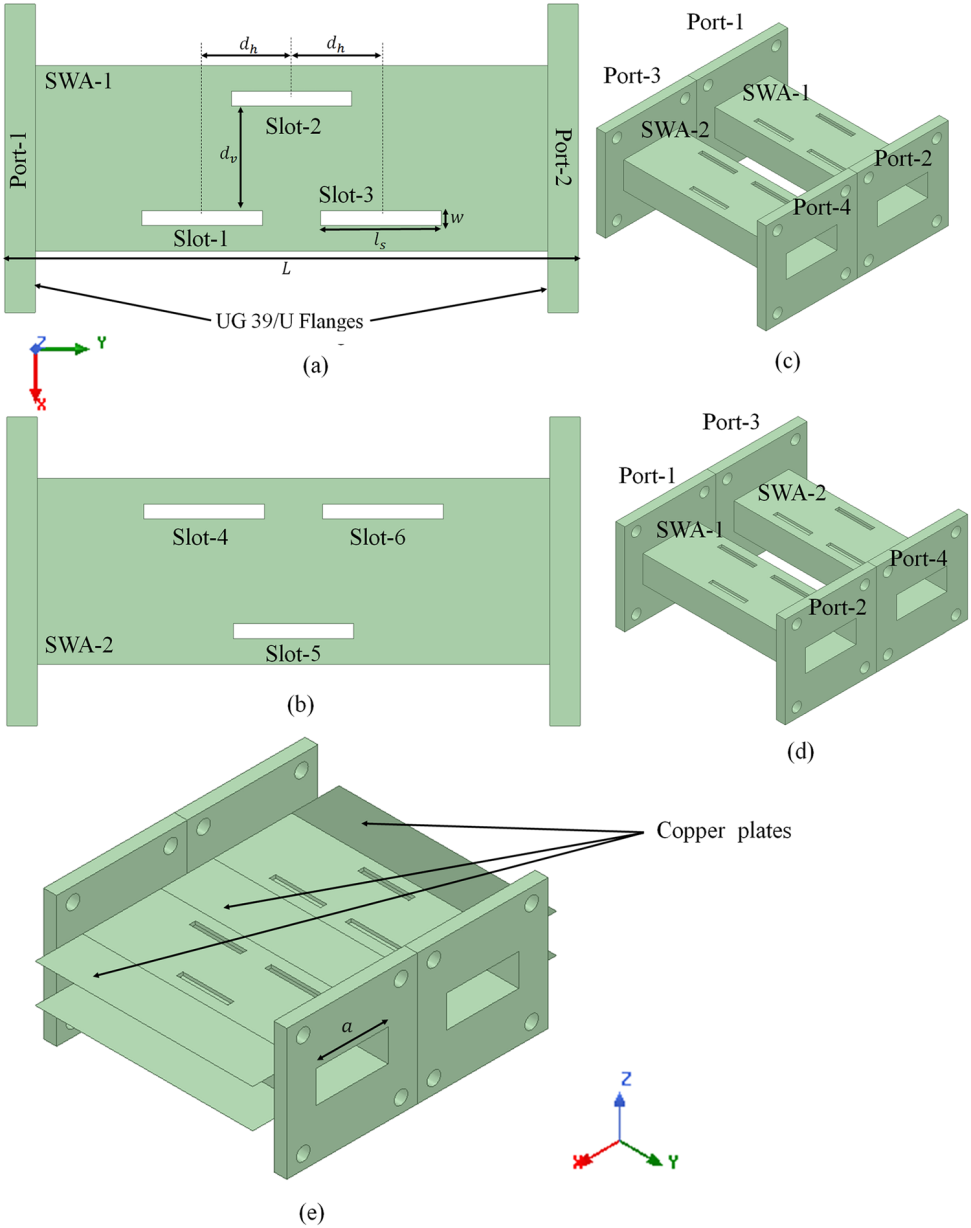
Geometry of the proposed SWA array, (**a**) top view of the SWA-1, (**b**) top view of the SWA-2, (**c**) isometric view of SWA array; top portion, (**d**) isometric view of SWA array; bottom portion and, (**e**) complete isometric view of proposed SWA array.

### Design of proposed SWA array

The geometry of the proposed slotted waveguide antenna array with a step-by-step design procedure is shown in Fig. 3. The conventional WR-90 rectangular waveguide is used to design the proposed SWA for X-band applications. As shown in Fig. 3a, the rectangular slots are made in both the top and bottom broad walls of a WR-90 waveguide at the same positions, i,e., these slots are made through the waveguide’s broad walls. Let the slots in the top broad wall be termed as Slot-1, Slot-2, and Slot-3 whereas in the bottom broad wall, be termed as Slot-1$^{\prime }$, Slot-2$^{\prime }$, and Slot-3$^{\prime }$. All these slots are placed judiciously in the broad wall to radiate with a required phase difference in the case of first-order OAM generation. The WR-90 waveguide used here is made up of copper material, and the walls’ thickness is kept at 1 mm.

The length, $l_{s}$ of the rectangular slots made in both the broad walls is 16.2 mm (i.e., $l_{s}\approx \lambda _{r}/2$), optimize for the resonating frequency ($f_{r}$) of 10 GHz. Here $\lambda _{r}$ is the wavelength corresponding to the resonating frequency, $f_{r}$. The width of the slot is 2 mm, optimized to have maximum radiation from these slots at 10 GHz. All the adjacent slots are placed at a horizontal distance of ($d_{h}\approx \lambda _{g}/4$), and the vertical distance of ($d_{v}=14$ mm), as shown in Fig. 3a, where the guided wavelength $\lambda _{g}$ can be calculated as,

4 $$\begin{aligned} \lambda _{g}=\frac{c}{f_{r}} \times \frac{1}{\sqrt{1-{(\frac{c}{2af_{r}})^{2}}}} \end{aligned}$$

where, *a* is the length of the WR-90 rectangular waveguide having typical value of 22.86 mm. The total length, *L*, of the SWA, including the flanges, is 77 mm, optimized for a good impedance match at a resonating frequency of 10 GHz.

After that, the SWA-2 is designed, where the slots made in both the top and bottom broad walls are swapped as compared to SWA-1, i.e., the slots in the upper half are shifted to the lower half of the broad wall and vice-versa. Let the slots of SWA-2 in the top broad wall be termed Slot-4, Slot-5, and Slot-6, whereas in the bottom broad wall, be termed Slot-4$^{\prime }$, Slot-5$^{\prime }$, and Slot-6$^{\prime }$. These two slotted waveguides, SWA-1 and SWA-2, are attached to form the array as shown in Fig. 3c, which shows the isometric view of an top broad wall, whereas Fig. 3d shows the isometric view of the bottom broad wall. Fig. 3e shows the complete view of the proposed SWA array where $\lambda /2$ wide copper plates with a thickness of 1 mm are attached to the top and bottom edges of the SWA array co-linear to the broad walls. These copper plates avoid the back radiation of the fields radiating from the slots improving the far-field realized gain. Moreover, these plates support the structural geometry of the antenna.

The EM simulations performed on the antenna structure verified that when the Port-1 and Port-4 of the proposed SWA array are excited with fundamental $TE_{10}$ mode, whereas Port-2 and Port-3 are matched terminated, $l=-1$ and $l=+1$ OAM modes are generated from the top and bottom broad wall of the waveguide, respectively. So the proposed antenna can simultaneously transmit the OAM beams with $l=-1$ and $l=+1$ modes in $+z$ and $-z$ directions, respectively. The corresponding phase values of the fields radiating from all the slots are shown in Table 2. The other port of the SWA is chosen to be matched terminated to avoid the reflections, which would re-excite the slots in a different phase and cause phase distortions in the generated OAM beams. The radiation characteristics, mode purity, and performance of the proposed SWA array are discussed in the following sections.

**Table 2:** Theoretical Phase values of E-field radiated by the slots of the proposed SWA array when Port-1 and Port-4: Input, Port-2 and Port-3: Matched Terminated.

$\text{Slot}$	$\text {Slot-1}$	$\text {Slot-2}$	$\text {Slot-3}$	$\text{Slot-4}$	$\text {Slot-5}$	$\text {Slot-6}$	$\text{OAM}$ $\text{mode}$
Radiated E-field phase	$0^{\circ }$	$-90^{\circ }$	$180^{\circ }$	$0^{\circ }$	$90^{\circ }$	$180^{\circ }$	– 1

$\text{Slot}$	$\text {Slot}-1{^prime}$	$\text{Slot}-2{^prime}$	$\text {Slot}-3{^prime}$	$\text {Slot}-4{^prime}$	$\text {Slot}-5{^prime}$	$\text {Slot}-6{^prime}$	$\text{OAM}$ $\text{mode}$
Radiated E-field phase	$0^{\circ }$	$90^{\circ }$	$180^{\circ }$	$0^{\circ }$	$-90^{\circ }$	$180^{\circ }$	+1

**Figure 4:**
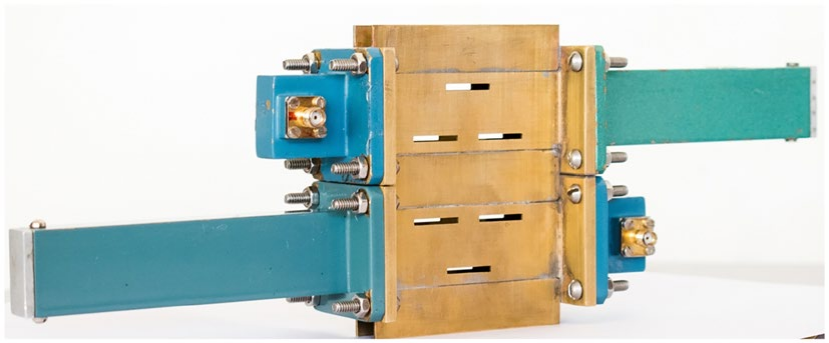
Fabricated prototype of the proposed SWA.

### Performance of the proposed SWA array

**Figure 5:**
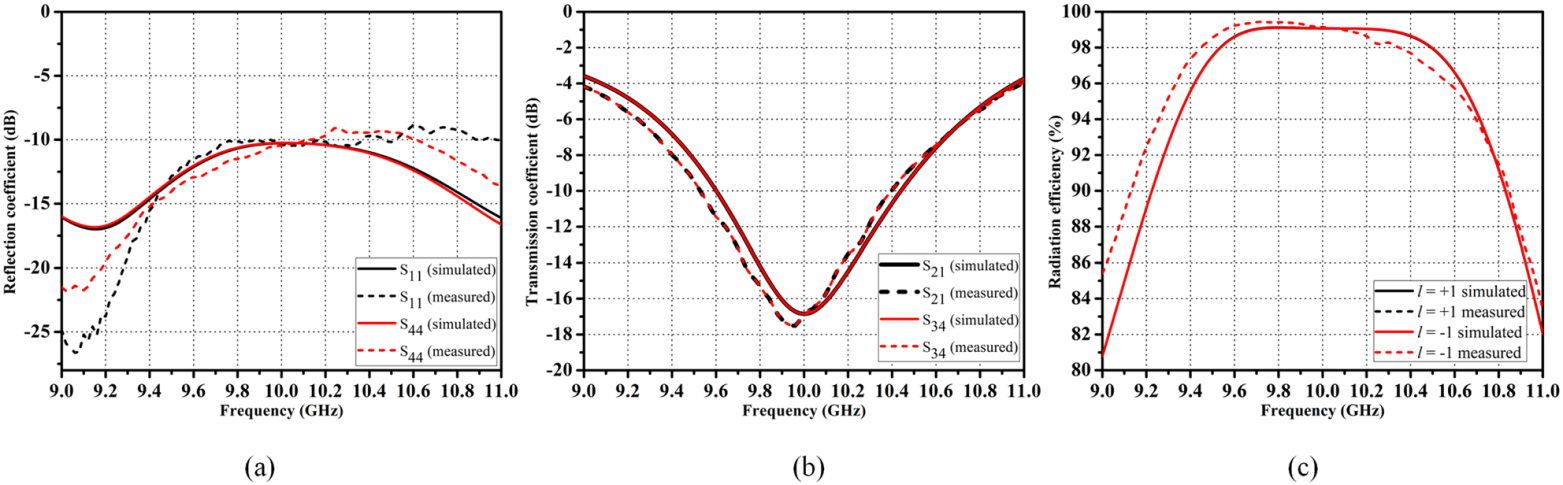
Scattering parameters and radiation efficiency plots of the proposed SWA, (**a**) reflection coefficient vs. frequency and, (**b**) transmission coefficient vs. frequency, and (**c**) radiation efficiency vs. frequency for both $l=\pm 1$ OAM modes.

Figure 4 shows the fabricated prototype of the proposed antenna with the excitation facilities at Port-1 and 4 and matched terminations at Port-2 and 3. In order to feed the structure, the SMA to waveguide adapters are used. Figure 5 shows scattering parameters characteristics along with radiation efficiency plot of the proposed SWA array. It can be observed in Fig. 5a that the proposed SWA has a reflection coefficient less than -10 dB at the frequency of 10 GHz, showing good return loss characteristics at the resonating frequency. It can be observed from the reflection curve that the proposed antenna has the percentage impedance bandwidth of almost around 20%. Also, Fig. 5b shows that the amplitude of transmission coefficient between Port-1 and 2 (i.e., $S_{21}$) and Port-3 and 4 (i.e., $S_{43}$) is less than -15 dB at 10 GHz, indicating the good amount of leakage from the designed slots at the resonating frequency. The measured scattering parameters agree well with the simulated ones. Here the scattering parameters are shown corresponding to Port-1 and 4 as input ports, and the other two ports are matched. But the scattering parameters by treating other ports as an input will also be the same because of the symmetry in the structure. Moreover, in case of SWAs, it is always important to compute the radiation efficiency in order to find the amount of leakage happening from the slots. Fig. 5c shows the radiation efficiency of the proposed antenna for both the +1 and –1 modes. It can be observed that the proposed SWA has around 99% efficiency at the resonating frequency 10 GHz. However, the percentage radiation bandwidth of the antenna is around 11% treating 95% as a reference.

**Figure 6:**
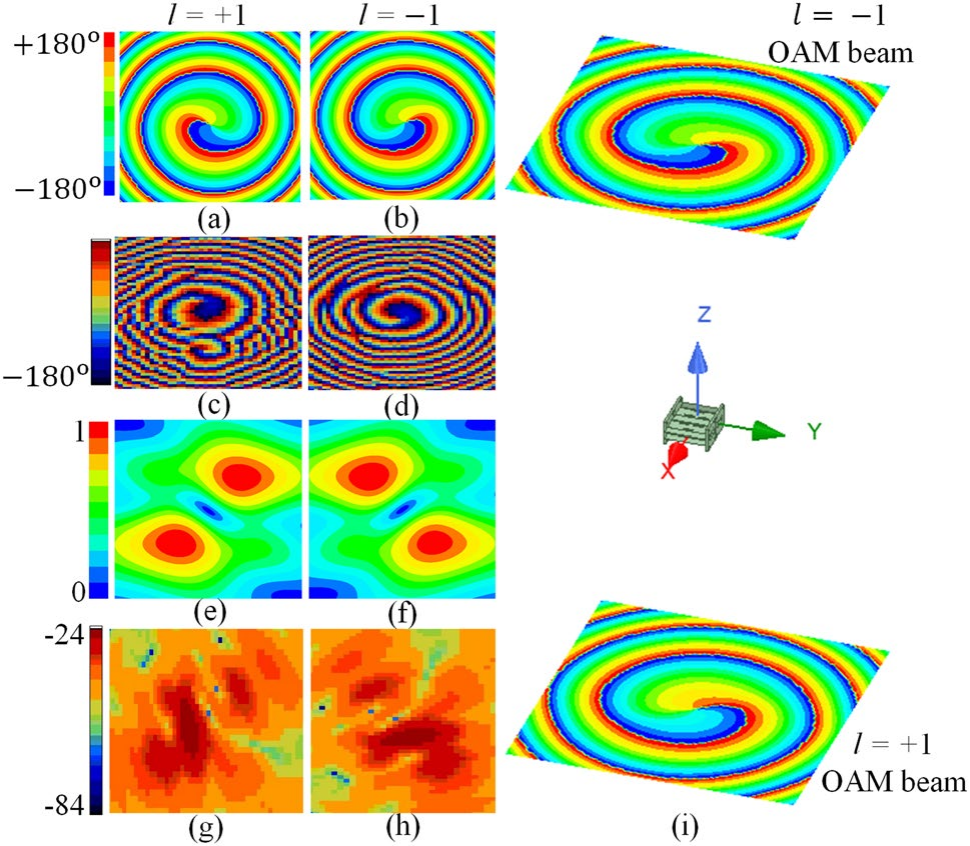
Near-field phase and amplitude distribution of the electric field at 10 GHz (**a**) simulated phase $l=+1$ mode, (**b**) simulated phase $l=-1$ mode, (**c**) measured phase $l=+1$ mode, (**d**) measured phase $l=-1$ mode, (**e**) simulated amplitude $l=+1$ mode, (**f**) simulated amplitude $l=-1$ mode, (**g**) measured amplitude $l=+1$ mode, (**h**) measured amplitude $l=-1$ mode, and (**i**) simultaneous OAM beams radiation scenario for both $l=\pm 1$ modes.

To confirm the OAM beam generation by the proposed SWA array, the near-field simulations followed by the near-field measurements are performed. Figure 6 shows the simulated and measured phase and amplitude distribution of the electric field at the resonating frequency of 10 GHz. When the proposed structure is fed, as shown in Fig. 4, the OAM beam with $l=-1$ mode is generated from the top slotted broad wall, as shown in Fig. 6b. At the same time, the OAM beam with $l=+1$ mode is generated from the bottom slotted broad wall, as shown in Fig. 6a. The conventional planar scanning method is used in the simulation to plot the phase and amplitude distribution of the electric field. The scanning windows of (500 mm $\times$ 500 mm) at a broadside distance of 540 mm are taken in the structure’s $+z$ and $-z$ directions. The clockwise and anticlockwise helical rotation of the E-field phase shown in the figure confirms the generation of $-1$ and $+1$ OAM beams, respectively. Moreover, the phase change from $-180^{\circ }$ to $180^{\circ }$ in one rotation confirms the first-order mode generation. In order to measure the near-field characteristics, the experiment is performed in the near-field anechoic chamber on the laboratory prototype. Figure 6c and d shows the measured phase distribution of the electric field for $l=+1$ and $l=-1$ modes, respectively, at 10 GHz. It can be seen that the measured phase plots are in good agreement with the simulated ones. The small distortion observed in the Fig. 6c is probably because of the reflections from SMA Tee used during the measurements. In addition, the simulated and measured near-field amplitude distribution of the generated OAM beams are shown in Fig. 6e,f. The amplitude null in the broadside direction as required by the OAM beams can be observed here. The measured amplitude plots agrees well with the simulated ones. The small variation in the amplitude plot especially in terms of null radius is because of the large opening of the probe and large scanning step size which is chosen as per the limitations of the experimental setup. Figure 6i shows the scenario of complete radiation from the proposed SWA array. It can be observed here that the proposed structure is radiating $-1$ and $+1$ OAM beams simultaneously in $+z$ and $-z$ directions, respectively.

**Figure 7:**
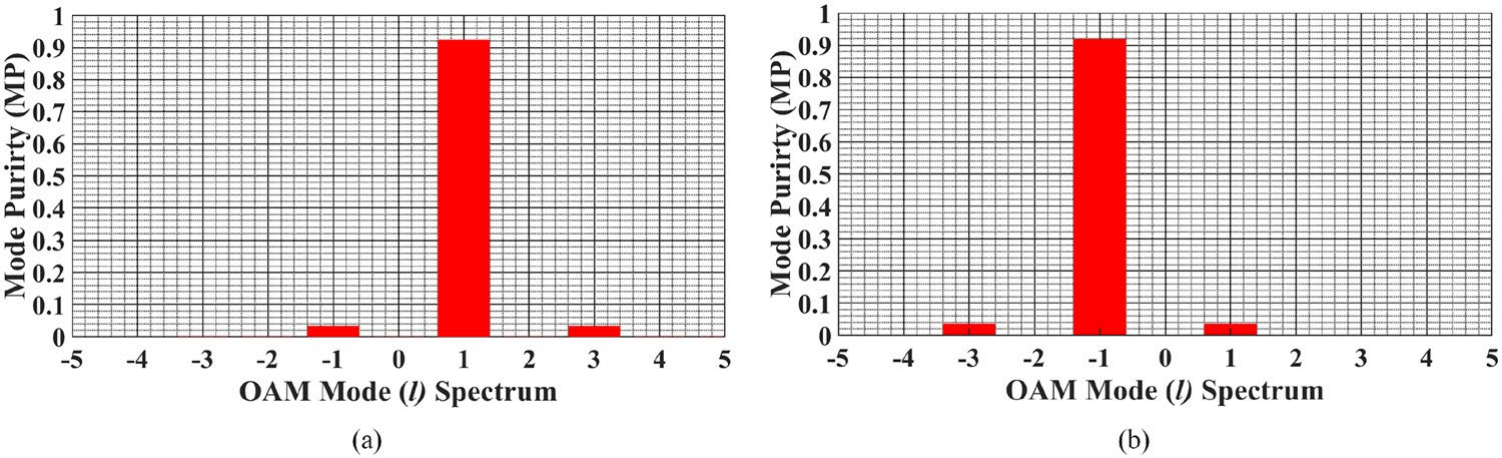
Mode purity spectrum of generated OAM beams at 10 GHz, (**a**) for $l=+1$ mode and, (**b**) for $l=-1$ mode.

In order to investigate the purity in the generated OAM beams, the mode purity analysis is performed, which further confirms the OAM beam generation by the proposed SWA array. The mode purity analysis can find the proportion of the energy in the designated mode over the entire mode spectrum. As the transverse azimuth axis ($\phi$) in Eq. (1) is periodic, the Fourier Transform of the same can give the modal spectrum of the OAM beams. In general, the mode purity analysis can be calculated by the spectral analysis of the following^28^,

5 $$\begin{aligned} \Psi (\phi ) = \frac{1}{\sqrt{2\pi }}\sum _{l = -\infty }^{\infty } A_{l} e^{jl\phi } \end{aligned}$$

6 $$\begin{aligned} A_{l} = \frac{1}{\sqrt{2\pi }} \int _{-\pi }^{\pi } \Psi (\phi ) e^{-jl\phi } d\phi \end{aligned}$$

where $\Psi (\phi )$ is angular distribution and $A_{\textit{l}}$ shows the distribution of angular momenta. Figure 7 shows the mode purity analysis of the generated OAM beams at the resonating frequency, calculated by solving Eqs. (5$)\,  \&  \,$(6) for the sampled field intensities points in the observation planes at near-zone. The analysis shows that the proposed SWA array can generate both $l=\pm 1$ modes with a good mode purity of 92$\%$, whereas a negligible power in the unintended modes can be observed.

**Figure 8:**
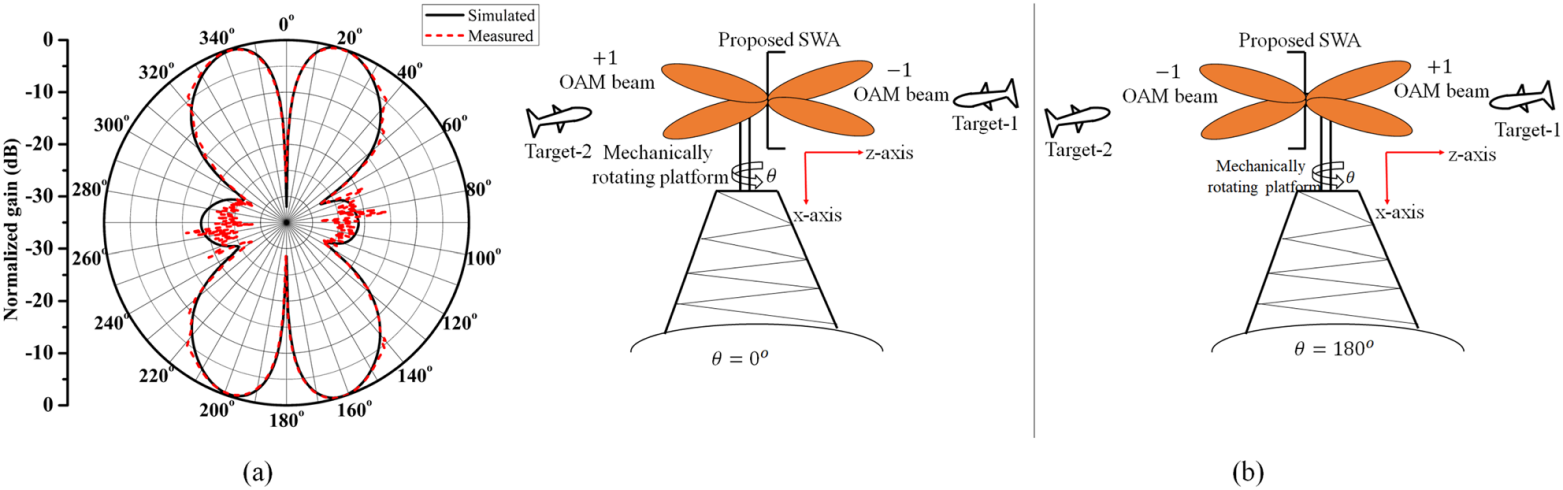
(**a**) Simulated and measured radiation pattern at 10 GHz, (**b**) scenario of proposed antenna mounted on mechanically rotating platform.

Figure 8a shows the simulated and measured far-field radiation pattern of the proposed SWA array, measured in a far-field anechoic chamber. The amplitude nulls can be observed at $\theta =0^{\circ }$ and $\theta =180^{\circ }$ i.e., along the $+z\,  \&  \,-z$ propagation axis, respectively, which satisfies one of the important properties of OAM beams called “phase singularity”. However, the divergence angle of the generated OAM beams is $\pm 18^{\circ }$. The good agreement between simulated and measured radiation pattern can be observed. It can also be observed that the proposed antenna structure is simultaneously radiating the OAM beams in both the upper (–1) and lower (+1) hemisphere, i.e., in $+z\,  \&  \,-z$ directions. So it can be said to have a quasi-omnidirectional radiation pattern, which can improve the coverage if the antenna is intended to be used for target detection purpose. The corresponding scenario is depicted in Fig.8b where the proposed antenna is assumed to be mounted on rotating platform. It can be seen here that because of its improved coverage, the proposed antenna can scan the areas on both sides simultaneously, which can reduce the probability of missing the target. Most importantly, it can also be observed that the same target is getting scanned by both the OAM modes when the platform is rotated by $180^{\circ }$, which can further improve the target resolution.

**Figure 9:**
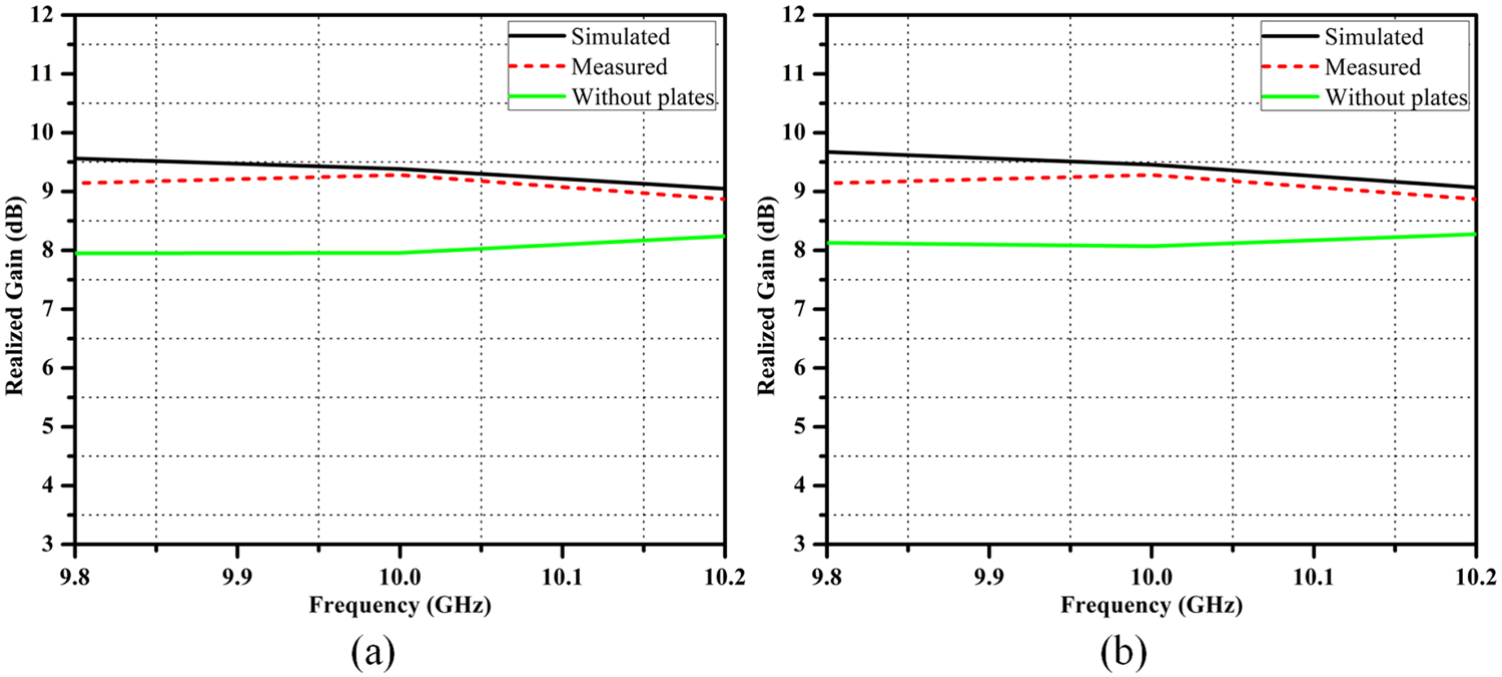
Simulated and measured far-field realized gain for (**a**) $l=+1$ mode, and (**b**) for $l=-1$ mode.

The far-field realized gain plots for $l=+1$ and $l=-1$ modes are shown in Fig. 9a and b, respectively. The proposed SWA array has a high gain of around 9.5 dB for both the OAM modes. The gain plots without the use of copper plates are also shown. It can be observed that the gain of the generated OAM beams improved by almost 1.5 dB after attaching the copper plates because, as discussed earlier, these plates reduce the back radiation of a particular mode and hence improves the gain. Table 3 shows the comparison analysis of the proposed SWA with the existing antennas that are frequently used for the OAM generation. It can be observed from the table that the OAM beams generated by the proposed antenna has the highest mode purity. Furthermore, the better performance in terms of feeding scheme, phase shifter requirement, and divergence angle can also be seen.

**Table 3:** Comparison study of the proposed SWA with an existing work.

Ref.	Antenna type	${\text{f}}_{\text{r}}$ (GHz)	Feeding scheme	Need of phase shifter	OAM modes	Gain (dBi)	Divergence angle	Mode purity	Power handling
^13^	Reflectarray	30	N.R.	N.R.	+ 2 and + 4	24	N.A.	$\sim$75$\%$	Low
^17^	UCA	5.5	Complex	Yes	+ 1 or $-1$	9.5, 10.2	$28^{\circ }$, $26^{\circ }$	N.A.	Low
^19^	UCA .	10.7	Simple	No	+ 1	N.A.	$60^{\circ }$	$\sim 50\%$	Less
^22^	CMT based patch	5.03	Complex	Yes	+ 3	6.89	$57^{\circ }$	Less	Low
^23^	Horn with SPP	7.4, 20, 40	Simple	No	+ 1 or $-1$	40, 30	$15^{\circ }$	$53\%$, $80\%$	High
^24^	Cassegrain reflector	18	Complex	Yes	+ 1 and $-1$	27.7	$3^{\circ }$	Very less	High
^29^	UCA	2.45	Complex	Yes	+ 1 or $-1$	7.1, 7	$\sim 35^{\circ }$	N.A.	Low
^30^	DRA	5.8	Complex	Yes	+ 1 and $-1$	5.4	N.A.	N.A.	Low
^31^	Helix	0.5-4	Simple	No	1–4	4.55	$30^{\circ }$	70$\%$, 80$\%$	Low
^32^	Leaky-wave SIW	11-12	Simple	No	+ 2 or + 1 or $-1$	7.18	N.A.	Less	Moderate
This work	Slotted waveguide	10	Simple	No	+ 1 and – 1	9.5	${\text{18}}^{\circ }$	92%	High

N.A. means Not Available, N.R. means Not Required.

## Conclusion

A simple slotted waveguide antenna array for generating first-order OAM beams has been designed and experimentally tested. The scattering parameters show that the proposed SWA operates well at a resonating frequency of 10 GHz and has a percentage impedance and radiation bandwidth of 20% and 11%, respectively. The simulated near-field characteristics, which are also experimentally validated, show that the proposed antenna structure can generate the OAM beams with $l=-1$ and $l=+1$ modes simultaneously from the top and bottom slotted broad walls, respectively. Comparison study shows the proposed antenna has all-metallic structure, handles high power, needs simple feeding scheme without any external phase shifter, and generates highly pure OAM beams over its counterparts. The far-field radiation characteristics show that the proposed SWA array has a high realized gain and quasi-omnidirectional radiation pattern. Also, the phase singularity in the generated OAM beams in the upper and lower hemisphere is verified. Because of its radiation characteristics, the proposed SWA array has an improved coverage compared to other OAM-generating structures. By mounting it on a mechanically rotating platform, the proposed antenna can be utilized in target-detecting applications.

Moreover, because of its high gain and high power handling, the proposed SWA array can mitigate the issues in long-distance OAM communication to a certain extent and can be utilized in strategic applications where the high-power microwave is involved. Presently, the generated OAM beams are limited to first-order modes, but the proposed approach opens up a new path towards generating OAM beams; however, higher-order modes generation can also be attempted.

## Methods

The slotted waveguide antenna array proposed in this paper is simple and easy to design and fabricate. The structure is designed, optimized, and simulated using Ansys High-Frequency Structure Simulator (HFSS) 2020 R2. All the simulation results, such as scattering parameters, near-field phase distribution, far-field radiation pattern, realized gain, and surface current distribution, is obtained from the HFSS. Firstly, a simple WR-90 rectangular waveguide is designed and simulated to propagate fundamental $TE_{10}$ mode. After that, the simple and conventional rectangular slots are made in both the broad walls of a waveguide. The positions of these slots in the broad walls are mainly optimized to get the radiation with the desired phase difference between the adjacent slots. The dimensions and positions of these slots are mentioned in the “Design of proposed SWA array” section. After that, UG 39/U flanges and 1 mm thick copper plates are attached to the SWAs. The dimensions of the flanges are taken from its datasheet.

The proposed SWA array is fabricated by mechanical fabrication procedures. The slots in the broad walls of a WR-90 copper waveguide are made by the milling process. The machining process on the waveguide walls makes the thickness of the walls 1 mm. Finally, the flanges and the copper plates are attached to the waveguide by the brazing process. In order to feed the structure, SMA to waveguide adapters operating in X-band are used, whereas the matched terminations are utilized to match the other ports. The scattering parameters at the ports are measured using Vector Network Analyzer, Agilent PNA-X N5247A.

The mode purity analysis on the simulation data is performed by using MATLAB R2021b, and the mode purity plots are obtained from the same. Firstly, the electric field distribution in the observation plane in the near-zone, as shown in Fig. 6e, is sampled circularly with a radius of $6\lambda _{r}$. Secondly, the spectral analysis on the sampling points is performed by using Eqs. (5) and (6). Finally, the mode purity is calculated by the ratio of power in the designated mode to the total power in the entire mode spectrum^33^.

**Figure 10:**
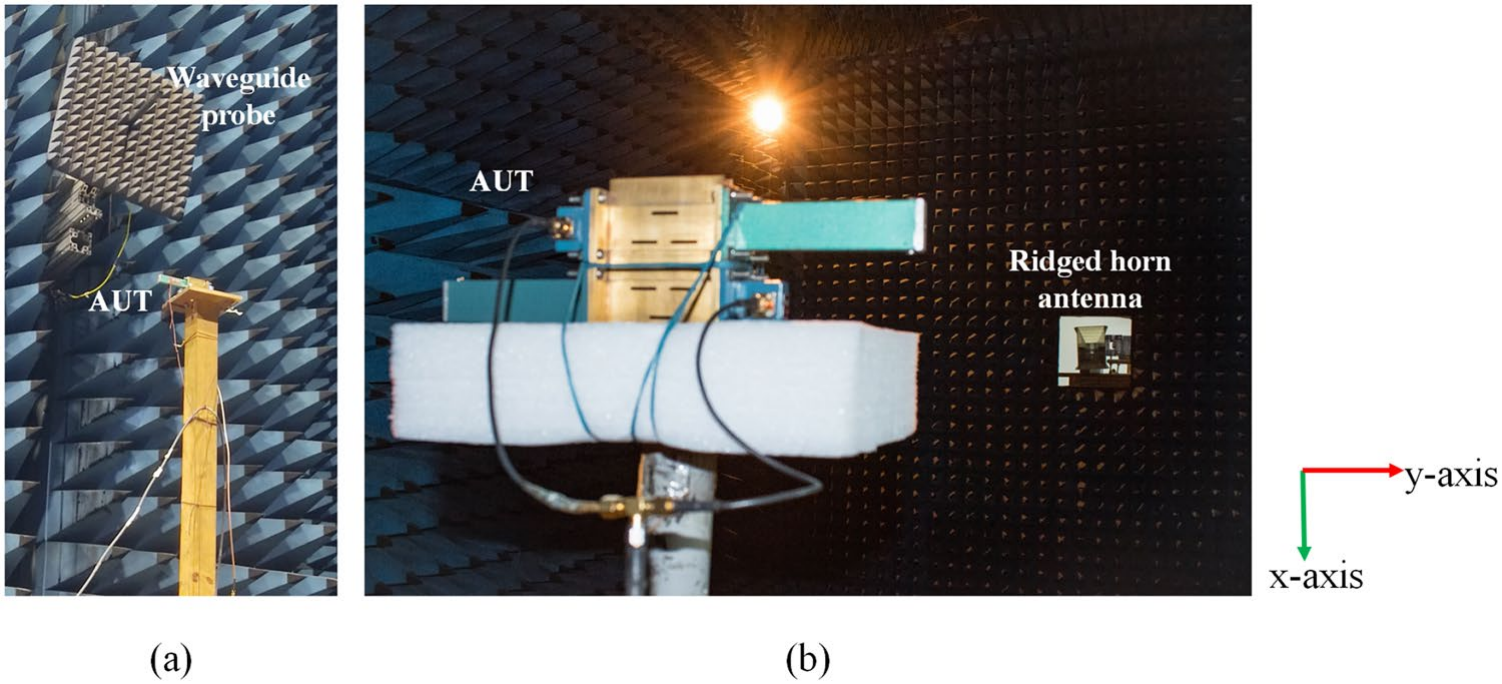
Measurement setup (**a**) near-field setup, and (**b**) far-field setup.

The near-field phase distribution of the electric fields generated by the proposed SWA array is measured in Near Field Test Range (NFTR) anechoic chamber. The experimental setup is as shown in Fig. 10a. The open-ended waveguide probe working in X-band is used on the transmitter side to scan the fields radiated by the Antenna Under Test (AUT). The probe used here is horizontally polarized, i.e., polarized in y-direction. This probe scans the planar area of (500 mm $\times$ 500 mm) at a distance of 180 mm from the radiating surface of the AUT. On the receiving side, two coaxial cables are connected to the SMA to waveguide adapters which are connected to Port-1 and Port-4. The SMA Tee is used to combine the power from these two coaxial cables. Firstly, the field in the $+z$ direction of the antenna is scanned, and then the field in the $-z$ direction is scanned.

The far-field radiation pattern and realized gain of the proposed SWA array are measured in the far-field anechoic chamber using the conventional methodology. The far-field experimental setup is shown in Fig. 10b. Note here that the ridged horn antenna used for the measurement purpose is also horizontally polarized, i.e., in y-direction. Here, the same cable connections are used on the receiving side, i.e., AUT, as used during the near-field measurements.

## Data Availability

All data generated or analysed during this study are included in this article.
